# A Minority of Desert Cyanobacteria and Algae Is Responsible for the Bulk of CO_2_ Fixation

**DOI:** 10.1111/ppl.70634

**Published:** 2025-11-12

**Authors:** Khin Maw Kyi, Mikhail V. Zubkov, Nina A. Kamennaya

**Affiliations:** ^1^ Kreitman School of Advanced Graduate Studies Ben‐Gurion University of the Negev Beer Sheva Israel; ^2^ French Associates Institute for Agriculture and Biotechnology, Blaustein Institutes for Desert Research Ben‐Gurion University of the Negev Midreshet Ben‐Gurion Israel; ^3^ Goldman Sonnenfeldt School of Sustainability and Climate Change Ben‐Gurion University of the Negev Beer Sheva Israel

**Keywords:** algae, biocrust, CO_2_ fixation, cyanobacteria, desert

## Abstract

Cyanobacteria and algae are the major photosynthetic organisms in deserts because they survive desiccation, high solar radiation and extreme temperature fluctuations better than other plants. Under favourable conditions, desert cyanobacteria and algae evidently photosynthesise. However, our understanding of whether each group modulates this metabolic process in response to preceding harsh conditions remains limited. To find out the effect of aridity on the photosynthetic activity of desert cyanobacteria and algae, we compared their cellular biovolume‐specific carbon dioxide (CO_2_) fixation in the hyper‐arid and arid regions of a typical hot desert—the central Negev Desert. We found that the biovolume‐specific CO_2_ fixation of both cyanobacteria and algae was highly variable rather than being constant. The values ranged from 20 to 40 folds, with 3–4 values out > 80 being similarly high. In response to aridity, at least a third of cyanobacteria and algae had low CO_2_ fixation activity (< 20% of the maximal rate) and contributed merely ~10% of the total CO_2_ fixation. Less than a quarter of cyanobacteria and algae fixed CO_2_ at a high rate (> 40% of the maximal rate) and, hence, were responsible for the bulk of CO_2_ fixation in the desert. Because the taxonomic composition of cyanobacterial and algal assemblages in the Negev is typical of biocrusts of the drylands, the aridity‐dependent dynamic of photosynthetic activity revealed in the Negev is likely a general trait for desert cyanobacteria and algae.

## 1 Introduction

Free‐living photosynthetic microscopic organisms—prokaryotic cyanobacteria and eukaryotic algae—thrive side by side in desert soils. In hyper‐arid and arid desert regions, they are considered the primary fixers of carbon dioxide (CO_2_) because they bear the harshest conditions, for example, intense solar radiation, extreme shifts in temperature and salinity and prolonged desiccation, better than most plants (Booth 1941; Cameron 1960; West 1990; Evans and Johansen 1999; Belnap and Lange 2001).

When moist and illuminated, desert photosynthetic microorganisms grow by fixing CO_2_ into their biomass. A fraction of the fixed carbon is exuded as carbohydrates, facilitating the development of a thin, mechanically relatively stable, biological crust (biocrust) on the soil surface above looser mineral substrate. Because in undisturbed areas biocrusts cover most of the open ground surface and mediate inputs, transfers and losses across the soil‐air boundary, they are key to desert soil stability and the functioning of the entire desert ecosystems (Belnap et al. 2016).

Moisture and sunlight are the primary growth drivers of the photosynthetic soil microorganisms (Belnap and Lange 2001; Belnap et al. 2004; Harel et al. 2004; Sponseller 2007; Büdel et al. 2009). Notably, the photosynthetic activity of intact biocrusts as well as of cyanobacterial and algal isolates from such biocrusts often reaches maximal levels at low‐to‐intermediate light intensities of 15–200 μmol photon m^−2^ s^−1^ and precipitation of 0.2–5.0 mm (Lange et al. 1992; Rao et al. 2009; Strong et al. 2013; Karsten et al. 2016; Zhang et al. 2018). This is probably because such mild conditions are typical for overcast rainy days or the first hours of daylight after night‐time dewfalls.

The durations and frequency of periods with the conditions that allow effective photosynthesis and other metabolic activities of photosynthetic soil microorganisms control the abundance of photosynthetic soil microorganisms and the developmental state of the biocrusts (Belnap 2006; Strong et al. 2013). As the aridity increases from semi‐arid through arid to hyper‐arid regions, biocrusts become thinner and their biomass decreases (Belnap 2006). Additionally, the taxonomic composition of biocrust microbial assemblages, generally, becomes less complex (Maestre et al. 2015; Muñoz‐Martín et al. 2019; Samolov et al. 2020), although the most stress‐tolerant cyanobacterial and algae persist. Considering the fundamental biological differences between cyanobacteria and algae, it remains unknown if the effect of aridity on oxygenic photosynthesis of desert cyanobacteria and algae—the central autotrophic metabolism in biocrusts of arid and hyper‐arid regions—differs or is similar.

Soil cyanobacteria and algae use oxygenic photosynthesis to assimilate inorganic carbon (HCO_3_^−^ and CO_2_) into organic matter, utilising the sunlight energy provided by light‐harvesting systems and releasing O_2_ as a by‐product (Hall and Rao 1999). Hence, CO_2_ and O_2_ dynamics above the soil surface can be used to estimate bulk photosynthetic activity of biocrusts, though such estimates are complicated by the fact that the CO_2_ and O_2_ dynamics integrate signatures of photosynthesis and respiration and are coupled to physicochemical processes (Šesták et al. 1971; Hoppert et al. 2004; Zhang and Zhang 2014; Witzgall et al. 2024). The photosynthetic activity of biocrusts can also be approximated using chlorophyll fluorescence that reflects the functional state of the light‐harvesting systems in photosynthetic organisms, albeit with limited spatial resolution (Schreiber et al. 1995; Green et al. 1998; Zhang and Zhang 2014; Mallen‐Cooper et al. 2020; Thomas et al. 2022) and without differentiating between cyanobacteria and algae. Finally, bulk photosynthetic CO_2_ fixation (Šesták et al. 1971; Beulig et al. 2015) and assimilation of carbon into specific macromolecules, for example, lipids and nucleic acids (Beulig et al. 2015; Andresen et al. 2018; Witzgall et al. 2024), can be measured using isotopic ^14^C or ^13^C tracers. However, the bulk tracer‐based measurements do not differentiate between cyanobacteria and algae, and carbon tracing methods do not address the effect of aridity on photosynthetic activities of soil cyanobacteria and algae.

Here, we took advantage of the high sensitivity of the ^14^C tracer and combined it with laser microdissection of autofluorescent photosynthetic microorganisms. Using the direct measurement of photosynthesis—incorporation of ^14^C‐labeled CO_2_ into cellular biomass of chlorophyll‐containing microorganisms—we compared photosynthetic activities of live desert cyanobacteria and algae as well as assessed photosynthetic heterogeneity within these two groups in response to aridity. Our aim was to compare the response of photosynthetic potentials of cyanobacteria and algae as two distinct groups of free‐living photosynthetic soil microorganisms rather than to assess the photosynthetic potential of their individual taxa. The latter task is beyond the scope of this study because it requires taxonomic identification of individual cells. Unfortunately, phylogenetically diverse desert cyanobacteria and algae are morphologically similar (Dvořák et al. 2017; Joseph and Ray 2024). Furthermore, in natural samples, they often lack the taxonomically informative features essential for their morphological identification. On the other hand, molecular identification of the microorganisms requires cell disruption, whereas an intact cell should be radio‐assayed to quantify how much ^14^C‐tracer the cell fixed. Hence, it is inconceivable to both identify and radio‐assay the same cell. Nevertheless, filamentous cyanobacteria and unicellular algae can be distinguished microscopically using chlorophyll autofluorescence that reveals their morphology and indicates their photosynthetic potential. This approach allowed us to compare the cellular biovolume‐specific photosynthetic activities of cyanobacteria and algae living side by side in desert soils.

To assess the effect of aridity on the photosynthetic activities of these two groups, we compared them in soils from the hyper‐arid and arid regions (aridity index [AI] = 0.02 and 0.06, respectively) of the central Negev Desert (Goldreich 2003; Kafle and Bruins 2009). We chose the Negev because it is a well‐studied hot desert generally representative of hyper‐arid and arid hot drylands (Hoffmann et al. 2012; Kidron and Starinsky 2012; Kidron and Temina 2013).

To characterise the soil biocrusts from the arid and hyper‐arid regions, we measured organic matter contents and chlorophyll pigment concentrations (as proxies for photosynthetic biomass) and assessed taxonomic composition of their cyanobacterial and algal assemblages. To reduce dependence of the measured parameters on particular landscape settings, which could affect metabolic activities in biocrusts (Hagemann et al. 2017; Wu et al. 2017; Zhou et al. 2020), we sampled soils from hill slopes and stream beds. To minimise the effect of soil heterogeneity, we collected samples from 10 to 20 random locations and pooled the collected samples together.

We incubated the soil samples, which originated from different aridity levels, under identical mild environmental conditions that resemble natural events of temporal moisture availability. We preincubated the soil samples in the dark for ~20 h to allow photosynthetic microorganisms to hydrate and recover. Then, the samples were incubated in a ^14^CO_2_‐amended atmosphere. After the incubation, the tracer‐labelled individual cyanobacterial filaments and algal cells were excised using a laser‐cutting microscope based on their morphology and chlorophyll fluorescence. Chlorophyll fluorescence was used as an indicator of photosynthetically active cells. The excised cells were radio‐assayed to measure the amounts of ^14^C‐tracer they assimilated.

## 2 Materials and Methods

### 2.1 Study Sites and Biocrust Sampling

Soil samples were collected in the Negev Desert from the hyper‐arid Ramon Crater (30°34′01′′–30°37′36′′ N, 34°55′16′′–34°43′46′′ E; AI = 0.02) (Goldreich 2003) on the 25th of January 2022 and 3rd of April 2023 and arid Halukim Ridge (30°51′15′′ N, 34°45′56′′ E; AI = 0.06) (Kafle and Bruins 2009) on the 26th of January 2022 and 6th of March 2023. Samples were collected following a rain (2.5 mm) or a dewfall event, respectively (Figure S1). In 2021/2022 and 2022/2023 rainy seasons, the hyper‐arid and arid regions received 46.0 mm and 65.6 mm (ng.fieldclimate.com) and 69.8 and 92.2 mm (Israel Meteorological Service) of precipitation, respectively.

2 mm–thick crusts were collected by driving a cut syringe into the surface soil to fill the 2 mm–thick space. Then, soil was supported from underneath using a wide spatula, lifted and transferred into a Petri dish. To account for landscape‐dependent heterogeneity, soil samples were collected from two locations on the north‐facing slope, from stream beds of three different streams in the hyper‐arid region, from the south‐facing and north‐facing hill slopes and from a stream bed in the arid region. To account for biocrust heterogeneity, samples were collected from 10 random points at each sampling location and amalgamated. The mixed soil samples were dried in a lab, sieved to remove stones and plant materials, and stored at ambient temperature and humidity for the following analyses.

### 2.2 Biocrust Organic Matter Content

The organic matter content in soil samples was measured using the rapid dichromate oxidation technique (Nelson and Sommers 1996). First, sieved and ground soil samples were oxidised with a potassium dichromate solution in the presence of concentrated sulphuric acid. Then, the remaining chromate was quantified by titration with ferrous ammonium sulphate. The soil contents of organic matter were calculated assuming a 76% recovery (Walkley and Black 1934). Three technical replicates were made for each sample from different locations, e. g., a slope or a stream bed.

### 2.3 Biocrust Chlorophyll Content

Chlorophyll *a* and *b* (Chl *a*, Chl *b*) pigments were heat‐extracted twice from ground soil samples using MgCO_3_‐saturated 100% ethanol. Absorbances at 665, 665, 649 and 750 nm were measured using a spectrophotometer (Varian Cary 50 Bio UV/VIS spectrophotometer) and concentrations of Chl *a* and Chl *b* were calculated following Ritchie (2006). Three technical replicates were made for each sample from different locations, for example, a slope or a stream bed.

### 2.4 Photosynthetic CO_2_ Fixation and Micro‐Dissection of Isotopically Labelled Cyanobacteria and Algae

To assess the capacity of cyanobacteria and algae to fix CO_2_, we incubated soil samples in air, in which ambient CO_2_ was replaced by radiolabelled carbon dioxide (^14^CO_2_) at a similar concentration. Weighed soil samples were dispensed in small plastic vessels, prewetted with distilled water under non‐photosynthetically active green light and kept in the dark overnight. The CO_2_ pulse that commonly occurred after soil rewetting in drylands (Sponseller 2007; Thomas et al. 2022) was vented. The next day, the vessels were moistened again to the equivalent of ~0.2–0.3 mm precipitation, which supported high rates of CO_2_ fixation (Lange et al. 1992) and allowed efficient gas diffusion. The vessels were placed in a humidified glass bottle with an ampule of radiolabelled bicarbonate (H^14^CO_3_^−^) solution, the bottle was sealed with a rubber septum lid and flushed through with moist CO_2_‐free air. Then, HPLC‐grade concentrated orthophosphoric acid was added to the ampule to release ^14^C‐labeled gaseous CO_2_ to the final partial pressure of ~400 ppm ^14^CO_2_. The experiment was set under non‐photosynthetically active green light. The experimental bottle was incubated at 65–70 μmol m^−2^ s^−1^ of white light (39 W LED, 6500 K, Osram) at ambient temperature for 45 h—time sufficient to detect ^14^C fixation in excised cells. After light incubation, soil samples were fixed with 2% paraformaldehyde (final concentration). Fixed samples were stored at +4°C in the dark to slow down pigment degradation.

**FIGURE 1:**
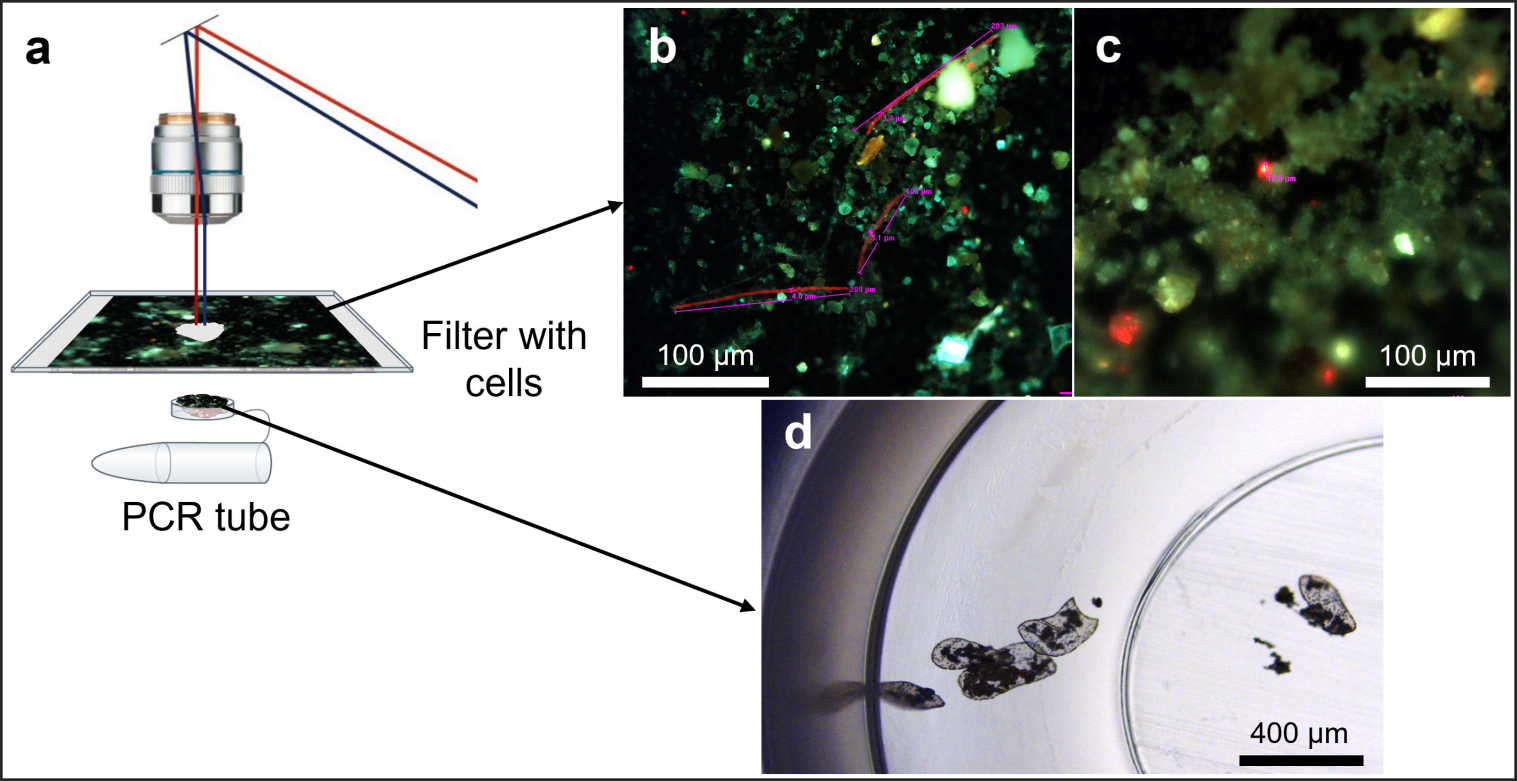
(a) Laser microdissection (LMD) microscopy was used to precisely excise (b) cyanobacterial filaments or (c) algal cells and (d) to deposit them in a cap of a PCR tube for downstream scintillation counting.

For microscopy, soil samples were dispersed in sterile water, deposited onto a filter membrane, washed to remove smaller contaminants and examined using long‐focus 20×, 40× and 63× dry objectives (Leica) in bright‐field and epifluorescence modes. Chlorophyll fluorescence was visualised using a Leica E4 filter system: BP 436/7, dichromatic mirror 455, suppression filter LP 470. For micro‐dissection, we selected cyanobacterial filaments or algal cells based on their chlorophyll autofluorescence, size, presence or absence of chloroplasts, and cell morphology (see representative micrographs at https://osf.io/ch4sf/?view_only=2b176b254ed74843afdc951ad727a858). Dimensions of each cyanobacterial filament or algal cell were measured using LMD Application Software that was calibrated using S1 Micrometer (Electron Microscopy Science). Then, the cells were micro‐dissected using a focused UV laser beam (LMD7, Leica) and gravity‐collected in a cap of a 0.2‐ml PCR tube filled with 50 μL of phosphate‐buffered saline (PBS, pH 9.0). The collection success was validated microscopically using a 5× long‐focus objective at the bright‐field and epifluorescent modes (Figure 1). On average, seven cyanobacterial filaments or eight algal cells were collected in each tube. As a negative control, similarly sized pieces of a membrane without cyanobacteria and algae were micro‐dissected and collected in tubes. The tube content was acidified with 250 μL of 1 M HCl and inorganic carbon was allowed to vent for 24 h. Following the removal of inorganic carbon, each tube was placed into a scintillation vial filled with 4 mL of ProSafe scintillation cocktail (Meridian). The ^14^C amount in excised cells was quantified as disintegrations per minute (DPM) using a triple‐PMT Hidex 300 Super Low scintillation counter (Hidex).

### 2.5 Statistical Analyses

One‐way analyses of variance (ANOVA) were used to compare the chlorophyll data sets to assess differences caused by heterogeneity in soil samples. After One‐Way ANOVA, the Tukey's HSD (honestly significant difference) test and the Tukey–Kramer test were used to compare the means. The logarithmic transformation was used when the variances of the data sets were larger than the means. When the data sets passed the Shapiro–Wilk test for normality, the two‐tailed *t*‐test was used to compare means. Differences were considered insignificant (=) at *p* > 0.05, statistically significant (*) at 0.005 < *p* < 0.05, highly significant (**) at 0.0005 < *p* < 0.005 and very highly significant (***) at *p* < 0.0005.

### 2.6 Molecular Characterisation of Cyanobacterial and Algal Assemblages

#### 2.6.1 Soil DNA Extraction

DNA was extracted from 6 g soil following the S protocol for soil DNA extraction (Sagova‐Mareckova et al. 2008) modified from Miller et al. (1999). The extracted DNA was further purified using the Quick‐Start DNeasy PowerSoil Pro Kit (Qiagen), and DNA concentration and quality were assessed using the NanoDrop ND‐1000 spectrophotometer (ThermoScientific Inc).

#### 2.6.2 PCR Amplification of 16S rRNA Gene

The 1st‐stage PCR amplification was carried out using high‐fidelity Phanta Flash master mix (Vazyme) and CYA‐359F and CYA‐781R cyanobacteria‐specific primers (Nübel et al. 1997) tailed with CS1 and CS2 adaptors for Amplicon Library preparation (Fluidigm, Access Array Barcode Library for Illumina; Naqib et al. 2018). Triplicate 10‐μL PCR reactions were performed along with the positive and negative controls, which contained either genomic DNA of cultured cyanobacteria or sterile water, respectively. PCR products were visualised using 2% agarose gel electrophoresis and successful reactions were pooled.

#### 2.6.3 Library Preparation and Sequencing

The pooled PCR products were sent to the Rush Genomics and Microbiome Core Facility (GMCF), where the 2nd‐stage PCR was performed in 10 μL reactions using repliQa HiFi ToughMix, with unique dual indices and 1 μL of the 1st stage PCR product was used as a template. PCR products were pooled and cleaned using Pippin Prep with 1.5% agarose gel, targeting DNA fragments (375–750 bp), followed by a 0.6× Ampure cleanup. Sequencing was done at GMCF using an Illumina MiniSeq with a 10% phiX spike‐in (2 × 154 paired‐end reads).

#### 2.6.4 Bioinformatic Analysis

The raw data were deposited to Illumina's BaseSpace platform as FASTQ files. Primer sequences and adapters were removed from the raw sequences using Cutadapt (Martin 2011). Quality filtering, denoising and removal of chimeric sequences were performed through the DADA2 pipeline, which clustered the sequences into amplicon sequence variants (ASVs) (Callahan et al. 2016). Taxonomy for the ASVs was assigned using the CyanoSeq (version 1.2) database (Lefler et al. 2023) for cyanobacteria and Greengenes2 (McDonald et al. 2024) and NCBI (Federhen 2012) for algae. Reads identified as Archaea or mitochondria were filtered out.

## 3 Results and Discussion

### 3.1 Biocrust Characterisation

**FIGURE 2:**
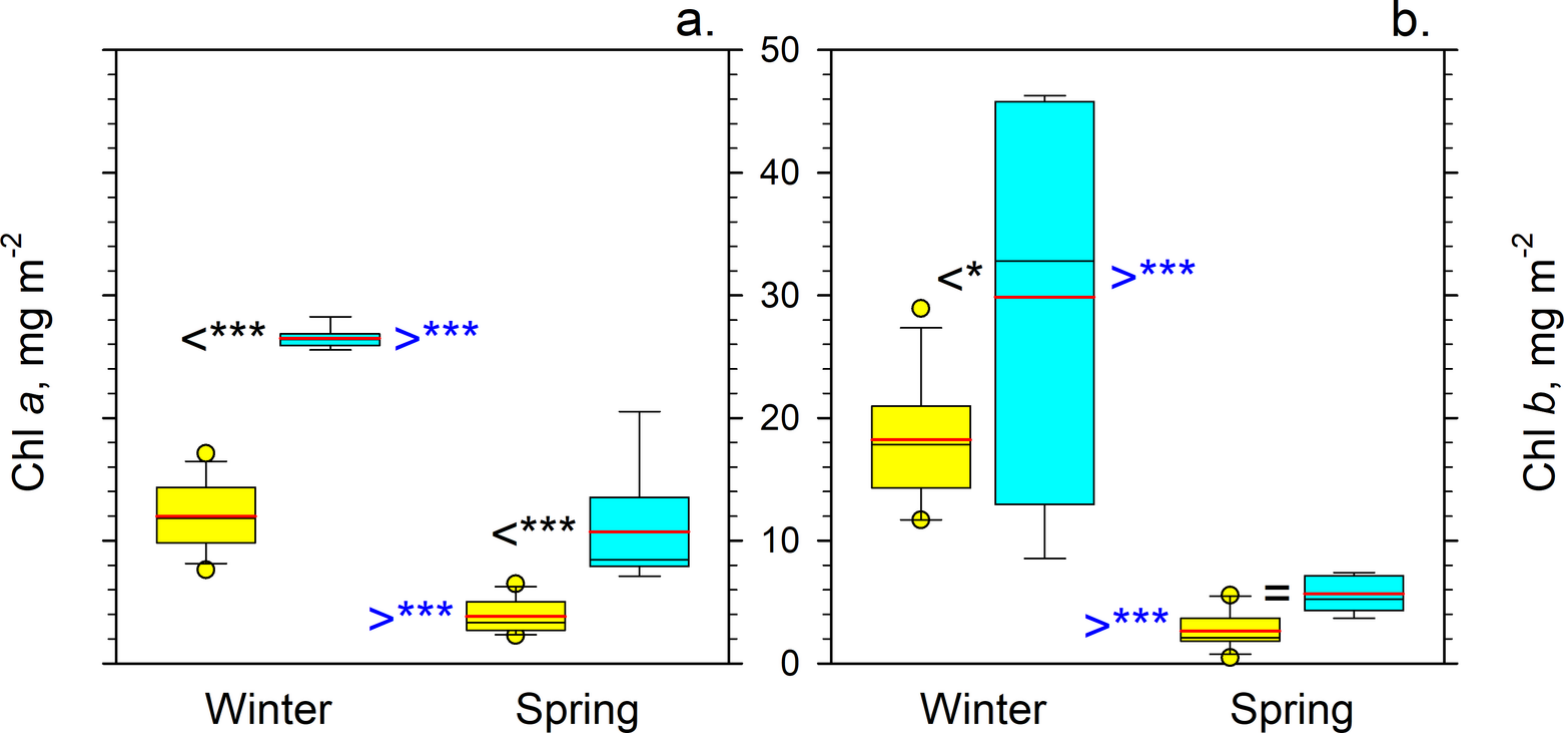
Comparison of (a) Chl *a* and (b) Chl *b* contents of the soil (mg m^−2^) from the hyper‐arid (yellow) and arid (cyan) regions collected in winter and spring. Box plots show the spread and skewness of data of the five (hyper‐arid) or three (arid) sampling locations with three technical replicates each. The medians and means are indicated by the black and red lines, respectively. The corresponding comparisons of the means using the Tukey's HSD tests are indicated by ‘<’, ‘=’ and ‘>’ symbols. The black symbols indicate comparisons between the hyper‐arid and arid regions. The blue symbols indicate comparisons between winter and spring for the arid or hyper‐arid region, respectively. Associated asterisks indicate the level of statistical significance: *, 0.005 < *p* < 0.05; ***, *p* < 0.0005.

The inference of high aridity at the central Negev desert was backed by low organic matter content of the biocrusts, which decreased from 1.56% ± 0.23% (*n* = 15) in the arid region to merely 1.23% ± 0.11% (*n* = 6) in the hyper‐arid region. Abundance of cyanobacteria and algae assessed by concentrations of photosynthetic pigments in biocrusts showed a similar trend: the Chl *a* and Chl *b* contents of the soil from the hyper‐arid and arid regions were significantly higher in winter than in spring (Figure 2). The Chl *a* contents were significantly lower in the hyper‐arid than in the arid region irrespective of the season, whereas the Chl *b* contents were more variable and less different. The mean concentrations of Chl *a* in the hyper‐arid and arid regions were 12.0 ± 2.7 and 26.5 ± 0.8 mg m^−2^ in winter; whereas, in spring, they were 3.8 ± 1.4 and 10.7 ± 4.5 mg m^−2^, respectively (Figure 2a). The mean concentrations of Chl *b* in the hyper‐arid and arid regions were 18.2 ± 5.1 and 29.9 ± 15.2 mg m^−2^ in winter; whereas, in spring, they were 2.6 ± 1.5 and 5.7 ± 1.5 mg m^−2^, respectively (Figure 2b). These chlorophyll concentrations were at the low end of the concentrations in other arid regions in the Negev (Kidron et al. 2010) and worldwide (Weber et al. 2018; Hui et al. 2021), indicative of severe moisture limitation of biocrust productivity. The significant difference in concentrations of Chl *a* and Chl *b* reflected the difference in the abundance of cyanobacteria and algae between the hyper‐arid and arid regions. However, this difference gave no insight into the ability of soil cyanobacteria and algae to modulate their photosynthetic activity in response to increasing aridity.

### 3.2 Comparison of Biovolume‐Specific CO_2_ Fixation by Micro‐Dissected Cyanobacteria and Algae

Micro‐dissection was the ultimate technique to test whether cyanobacteria and algae from the hyper‐arid and arid regions had similar or varied photosynthetic activities. Microscopic examination of the soil revealed the presence of filamentous cyanobacteria, rarely with heterocysts, and chloroplast‐bearing algae, mostly as single cells, and only occasionally in small clusters. No filamentous algae were found in the soil samples examined. For samples collected in winter, fewer cyanobacteria and algae were found and micro‐dissected in the hyper‐arid region than in the arid region (Figure S2). When we repeated the ^14^CO_2_ fixation and micro‐dissection experiment in spring, an even lower abundance of cyanobacteria and algae in the hyper‐arid region (Figure 2) made their micro‐dissection impractical. Therefore, here we compare the results of CO_2_ fixation by cyanobacteria and algae from the hyper‐arid region in winter with the results from the arid region in winter and spring.

**FIGURE 3:**
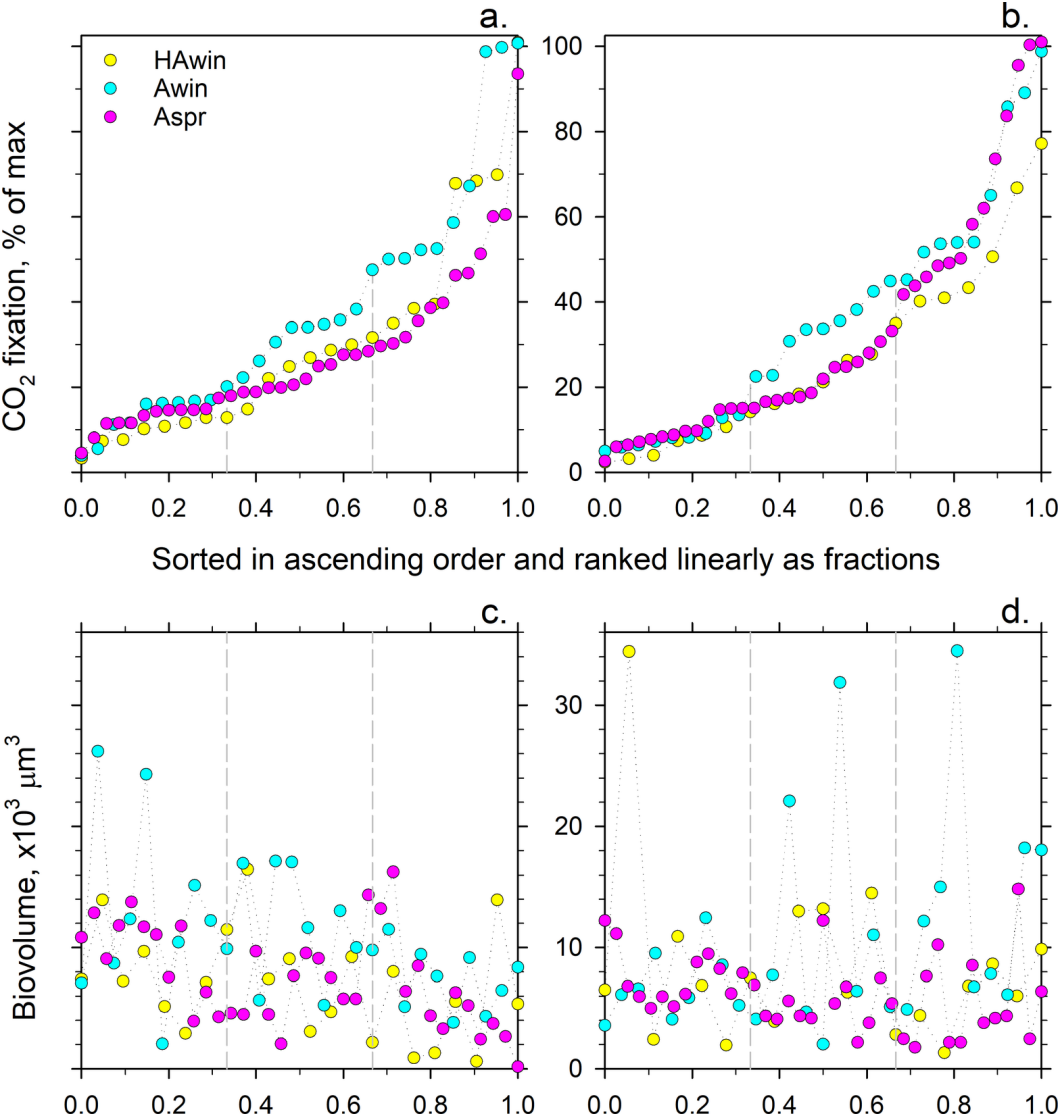
Relative biovolume‐specific CO_2_ fixation of soil (a) cyanobacteria and (b) algae. Dash lines delineate the three thirds of the *x*‐axis, which represent the ‘low‐’, ‘medium‐’ and ‘high‐activity’ parts (see in the text for the details). The corresponding biovolumes of biocrust (c) cyanobacteria and (d) algae are presented for comparison with CO_2_ fixation.

We found weak correlation and large scatter of CO_2_ fixations versus corresponding biovolumes of cyanobacteria or algae, irrespective of a region or a season (Figures 3 and Figure S2). This finding was surprising because all cells were excised based on red chlorophyll autofluorescence and therefore were anticipated to be photosynthetically active (see ‘Micrographs’ in Supporting Information: https://osf.io/ch4sf/files/osfstorage?view_only=2b176b254ed74843afdc951ad727a858). Because biovolume estimates were based on cellular dimensions measured microscopically, we checked the reproducibility of the dimension measurements. The mean diameters of the excised algal cells and the mean widths of the excised cyanobacterial filaments were compared in five and six independent experimental batches, respectively (Table S2). Because no statistically significant differences were found between the means of any pair, we consider the reproducibility of microscopic measurements adequate for the task. Therefore, the weak correlation between the CO_2_ fixations and the corresponding biovolumes (Figure S3) indicated that photosynthesis of both cyanobacterial and algal cells was variable rather than constant. To proceed with data analysis, we transformed the raw measurements of CO_2_ fixations into the CO_2_ fixation per unit of biovolume, that is, divided CO_2_ fixation by biovolume. The 20–40 times variance of the biovolume‐normalised values indicated the presence of cells with wide‐ranging photosynthetic CO_2_ fixation activities.

The four highest values (out of 86) of the biovolume‐specific CO_2_ fixation for cyanobacteria (one derived from the hyper‐arid region, three from the arid region in winter and none from the arid region in spring) were remarkably similar, that is, their coefficient of variance (CV) was merely 1.0%, whilst the other values were significantly lower. The three highest values (out of 85) of biovolume‐specific CO_2_ fixation for algae (none from the hyper‐arid region, one derived from the arid region in winter and two from the arid region in spring) were also very similar, that is, their CV was 1.2%. Such low variance between the highest values was interpreted as the maximal biovolume‐specific CO_2_ fixation achieved by the cyanobacteria and algae. In other words, all cells within the excised cyanobacterial filaments and all chloroplasts in the excised algal cells were photosynthetically highly active, that is, had the highest biovolume‐specific CO_2_ fixation. Henceforth, these values will be referred to as ‘the maximal values’.

All other biovolume‐specific values determined were then divided by the maximal values, that is, transformed into percentages of the maximum. To aid comparison between regions and seasons the percentages were sorted and presented in ascending order from the minimal value to 100%, that is, the maximal values (Figure 3a,b). Finally, the percentages were evenly distributed in ascending order between 0 and 1 along the abscissa (*x*‐axis, Figure 3a,b) to account for the differences in the number of micro‐dissected samples from each site (Figure S2). The above transformations resulted in easier‐to‐compare profiles of biovolume‐specific CO_2_ fixation determined for soil cyanobacteria or algae from different locations and seasons (Figure 3a,b).

The profiles revealed clear sections of similarities as well as differences in distributions of relative cyanobacterial and algal CO_2_ fixation (Figure 3a,b), which showed no relation to the corresponding biovolumes (Figure 3c,d). Relative CO_2_ fixations were distinctly different, whereas the mean biovolumes were statistically similar in the separated fractions, that is, photosynthetic activity was unrelated to biovolumes.

Irrespective of the region or season, at least the 0.33 fraction (*x*‐axis) of cyanobacteria and algae had CO_2_ fixation below 20% of the maximal values (Figure 3a,b; *y*‐axis). In the middle region of 20%–50% of the maximal CO_2_ fixation (*y*‐axis), the CO_2_ fixation of cyanobacteria and algae collected in winter from the arid region (Figure 3a,b; cyan) was higher compared to the values for cyanobacteria and algae from other regions or seasons (magenta and yellow). The CO_2_ fixation of the remaining cyanobacteria and algae was more than half of the maximal values (Figure 3a,b). These highly active organisms accounted for a fraction from 0.67 to 1 (*x*‐axis) of cyanobacteria from the arid region in winter (Figure 3a, cyan) and algae from the arid region in winter and spring (Figure 3b; cyan and magenta). Only a fraction from 0.8 to 1 (*x*‐axis) of cyanobacteria and algae from the hyper‐arid region in winter (Figure 3a,b; yellow) and cyanobacteria from the arid region in spring (Figure 3a, magenta) was highly active.

To generalise the profile interpretation (Figure 3a,b), we formally divided the profiles into three equal parts (0–0.33, 033–0.67 and 0.67–1 fractions on the *x*‐axis) and referred to these parts as ‘low‐’, ‘medium‐’ and ‘high‐activity’ parts. Assessment of the significance of the differences within each part separately is presented in Figure S3.

### 3.3 Comparison of the Integrated CO_2_ Fixation by Cyanobacterial and Algal Assemblages in the Hyper‐Arid and Arid Regions

**FIGURE 4:**
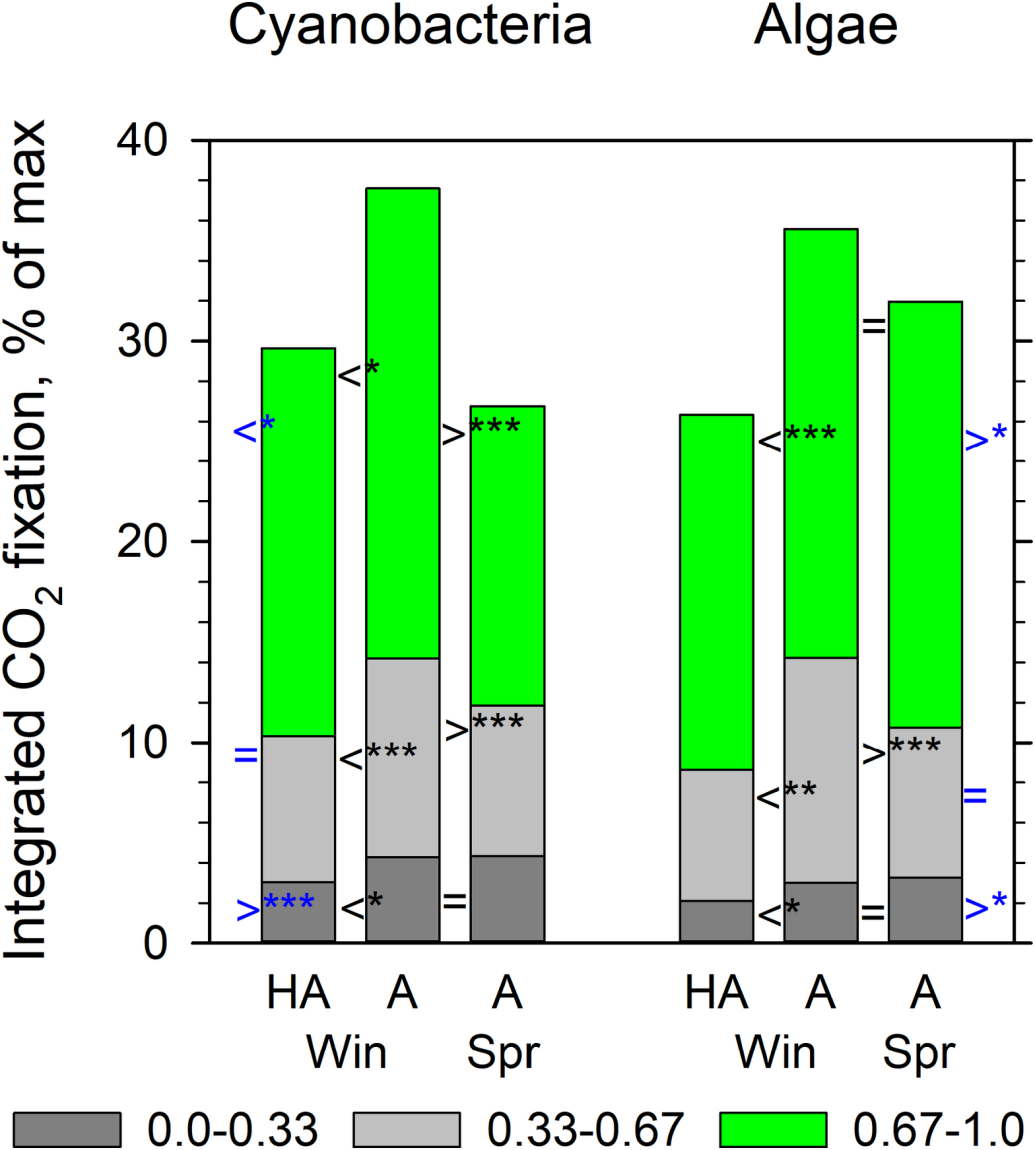
The percentage of integrated maximal CO_2_ fixation by the entire assemblage of soil cyanobacteria or algae from the hyper‐arid (HA) and arid (A) regions in winter (Win) and spring (Spr). Dark grey, light grey and green colours of the bar segments represent low‐, medium‐ and high‐activity parts. Statistically significant differences between the activity parts are marked with ‘>’ and ‘<’ symbols. The ‘=’ symbol marks statistically similar activities. Comparisons between the HA and A in spring are shown by blue symbols to the left of the HA for cyanobacteria and to the right of A Spr for algae. Associated asterisks indicate the level of statistical significance: *, 0.005 < *p* < 0.05; **, 0.0005 < *p* < 0.005; ***, *p* < 0.0005.

To quantitatively compare the photosynthetic activities of soil cyanobacteria and algae from regions with different aridity levels (Figure 3a,b), we calculated the areas under the complete CO_2_ fixation profiles, that is, computed the integrals for each of the profile curves. The resulting values of integrated CO_2_ fixation presented the percentages of the maximal (100%) CO_2_ fixation by the entire assemblage of cyanobacteria or algae (Figure 4, bar heights). Additionally, we colour‐labeled the relative contributions to the integrated CO_2_ fixation of the low‐, medium‐ and high‐activity cyanobacteria or algae (Figure 4, dark grey, light grey and green bar segments). The significance of the differences for the parts (Figure S3, box plot inserts) remained valid for the areas and is presented in Figure 4 as symbols.

In winter, the 38% and 36% integrated CO_2_ fixation by the arid‐region cyanobacterial and algal assemblages, respectively, was closer to their potential maximum of 100% compared to the 30% and 26% integrated CO_2_ fixation by the assemblages from the hyper‐arid region (Figure 4; Win, HA vs. A). The difference in CO_2_ fixation was significant for low‐, medium‐ and high‐activity parts. In spring, CO_2_ fixation by the soil algal assemblage in the arid region remained similar to that in winter, whereas CO_2_ fixation by the cyanobacterial assemblage decreased owing to the significant decrease in the high‐activity part of the assemblage (Figure 4, green).

**FIGURE 5:**
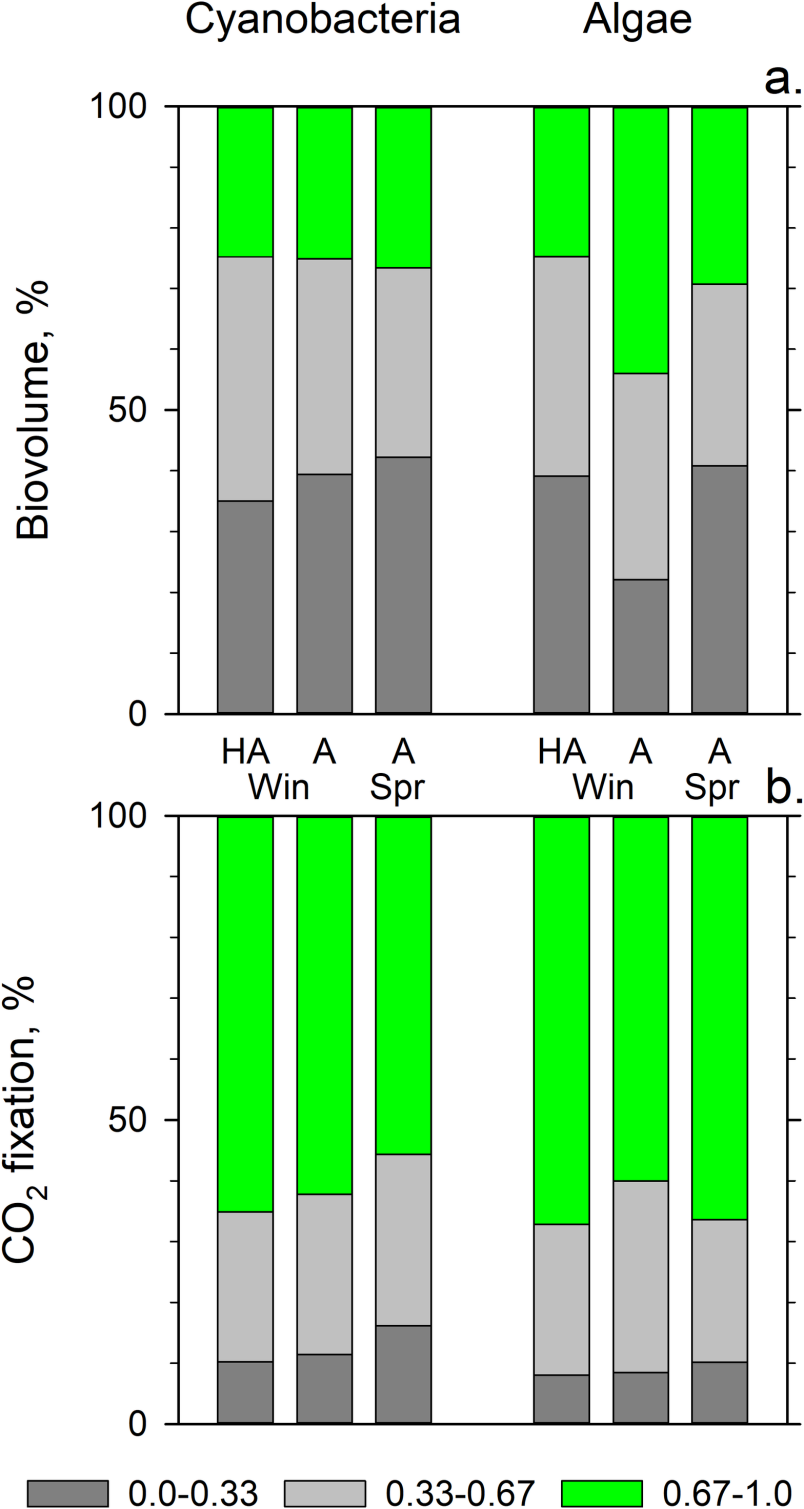
Comparison of (a) percentages of the total biovolume of the low‐ (dark grey), medium‐ (light‐grey) and high‐ (green) activity parts of the soil cyanobacterial or algal assemblages and (b) percentages of their contribution to the total CO_2_ fixation by the cyanobacteria and algal assemblages from the hyper‐arid (HA) and arid (A) regions in winter (Win) and spring (Spr).

To emphasise the relative contribution of the low‐, medium‐ and high‐activity parts to the CO_2_ fixation by a cyanobacterial or algal assemblage, we calculated the percentage of the total cellular biovolume of cyanobacterial and algal assemblages represented by each of the activity parts. To calculate that, we summed the biovolumes of cyanobacteria or algae with corresponding activities (Figure 3c,d) and divided them by the total biovolume of all cyanobacteria or algae analysed (Figure 5a). Then, we calculated the relative contribution of each of the fractions (Figure 4, bar heights) to the integrated CO_2_ fixation by the cyanobacterial and algal assemblages analysed (Figure 4, dark grey, light grey and green segments).

When we compared the biovolume percentages of each of the activity fractions (Figure 5a) to the percentages of their contributions to the total CO_2_ fixation (Figure 5b), we found that cyanobacteria and algae of the low‐activity part (Figure 5, dark grey) contributed similarly low, < 11% and < 8.5% of total CO_2_ fixation in the hyper‐arid region and arid region, respectively (Figure 5b). These cyanobacteria and algae with low activity comprised ~40% of total biovolume in both regions and seasons examined. The only exception was in the arid region in winter, when algae with low activity comprised only 20% of the total algal biovolume (Figure 5a). Microorganisms with medium activity (Figure 5, light‐grey) comprised 30%–40% of the total biovolume (Figure 5a) while contributing 23%–32% to the total CO_2_ fixation (Figure 5b). Microorganisms from the high‐activity part (Figure 5, green) comprised 25%–29% of the total biovolume (Figure 5a), irrespective of the region or season, and contributed 56%–67% to the total CO_2_ fixation (Figure 5b), except for algae from the arid region that comprised 44% of the total biovolume (Figure 5a) but contributed to ~60% of the total CO_2_ fixation (Figure 5b). Cyanobacteria and algae with low and medium activity comprised together 71%–75% of the total biovolume while contributing 33%–44% to the total CO_2_ fixation (Figure 5).

Therefore, most soil cyanobacteria and algae in the hyper‐arid and arid regions had low to medium photosynthetic activity. Because, in photosynthetic microorganisms, the rate of photosynthesis directly affects the rate of cellular growth, the growth rate of most soil cyanobacteria and algae in the hyper‐arid and arid regions was lower than the potential growth maximum. In heterotrophic bacteria, a sub‐population with low metabolic activity and a slow growth rate can evade antibiotic control (Balaban et al. 2004). This characteristic has been proposed as an adaptation to fluctuating environments (Balaban et al. 2004). Likewise, we interpreted that the cohorts of photosynthetic cells with low metabolic activity could be less affected by the aridity stress of the hyper‐arid and arid environments and, hence, more resilient. Finally, the cyanobacteria and algae with higher activity are a minority but are responsible for the bulk of CO_2_ fixation (Figure 5, green). Subsequently, minor fluctuations in their abundance caused by aridity could result in significant fluctuations in the total CO_2_ fixation in the desert.

### 3.4 Taxonomic Diversity of Cyanobacterial and Algal Assemblages

**FIGURE 6:**
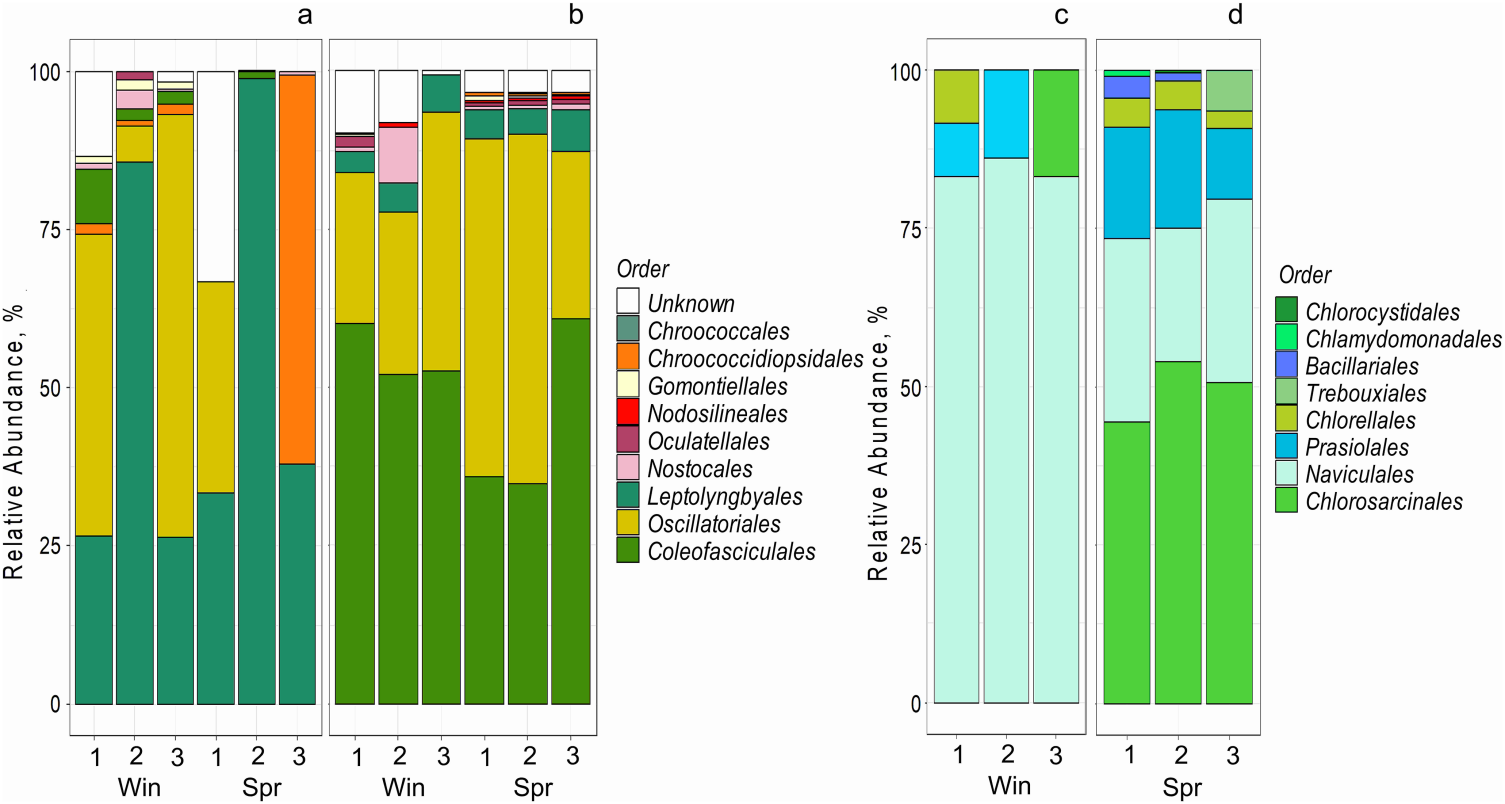
Relative abundance of cyanobacteria (a, b) and algae (c, d) in soils from the hyper‐arid (a, c) and arid (b, d) regions in winter (Win) and spring (Spr).

The observed variations in photosynthetic activity of both cyanobacteria and algae could be related to their taxonomy. To compare the taxonomic diversity of cyanobacterial and algal soil assemblages in the hyper‐arid and arid regions, we analysed 16S rRNA fragments of cyanobacterial genomes and algal chloroplast genomes. The variability in composition of cyanobacterial and algal assemblages between replicate samples of biocrusts from the hyper‐arid region (Figure 6a,c) was high, whereas the taxonomic composition of replicates was more reproducible in soil from the arid region (Figure 6b,d), suggesting that microbial surface distribution was patchier in the hyper‐arid region. The diversity of cyanobacteria in soils collected from the hyper‐arid region was slightly lower compared to the soil from the arid region. This is in agreement with the decreasing diversity of phototrophic microbial assemblages with the increasing aridity (Maestre et al. 2015). Specifically, cyanobacteria from 15 families (Figure S4) from seven orders (Figure 6a) were found in soil from the hyper‐arid region compared to 17 families (Figure S4) from nine orders in soils from the arid region (Figure 6b). In addition to the seven orders found in soils from both regions, *Chroococcales* and *Nodosilineales* orders were identified in the arid region only, but their relative abundance was low. Soils from the hyper‐arid region were dominated by *Leptolyngbya*, *Oscillatoriales* and, occasionally, *Chroococcidiopsidales*. The presence of *Coleofasciculales* and *Gomontiellales* in soils from the hyper‐arid region was notable mostly in winter. Soils from the arid region were dominated by *Coleofasciculales* and *Oscillatoriales*, whereas the abundance of the latter increased in spring.

The taxonomic diversity of algae in soils from the hyper‐arid and arid regions was restricted to four classes: one class of diatoms (Bacillariophyceae) and three classes of green algae (*Chlorophyceae*, *Trebouxiophyceae* and *Ulvophyceae*). Diatoms, mostly *Naviculales*, comprised > 75% of the sequences in the hyper‐arid region in winter (Figure 6c). In spring, *Chlorophyceae* (mostly *Chlorosarcinales*) comprised 45%–54% of the identified algae, followed by *Bacillariophyceae* (*Naviculales*) and *Trebouxiophyceae* (*Prasiolales*). The abundance of *Ulvophyceae* found in a single sub‐sample was very low (Figure 6d).

Lineages of both cyanobacteria and algae identified in soils from the hyper‐arid and arid regions are either common members of desert biocrust assemblages (e.g., *Leptolyngbyales*, *Oscillatoriales*, *Chroococcidiopsidales* and *Coleofasciculales* cyanobacteria and *Naviculales*, *Chlorophyceae* and *Trebouxiophyceae* algae) (Belnap et al. 2016) or have been recently found in the desert environments, e.g., *Nodosilineales* (Strunecký et al. 2023) and alga *Ulvophyceae* (Darienko and Proeschold 2017). Interestingly, the profiles of biovolume‐specific CO_2_ fixation determined for soil cyanobacteria or algae from different locations and seasons (Figure 3a,b) were relatively similar, that is, comprised comparable percentages of low‐, medium‐ and high‐activity cohorts, even when the taxonomic composition of cyanobacteria was dramatically different (Figure 6). For instance, the biovolume fractions and photosynthesis contribution of low‐, medium‐ and high‐activity cohorts of cyanobacteria from the hyper‐arid and arid region in winter (Figure 5) were remarkably similar, though in the hyper‐arid region the cyanobacterial assemblage was co‐dominated by *Leptolyngbyales* and *Oscillatoriales* compared to the arid region where *Coleofasciculales* dominated over *Oscillatoriales* (Figure 6a,b). Similarly, the biovolume fractions and photosynthesis contribution of low‐, medium‐ and high‐activity cohorts of algae from the hyper‐arid region in winter and the arid region in spring (Figure 5) were similar, whereas *Naviculales* strongly dominated the former algal assemblage and *Chlorosarcinales* dominated the latter (Figure 6c,d). Therefore, it is likely that the physiological rather than taxonomic heterogeneity of cells could be responsible for different levels of photosynthetic activity within the low‐, medium‐ and high‐activity cohorts of cyanobacteria and algae (Figures 4 and 5). If this explanation is correct, the low‐activity cohorts of cyanobacteria and algae could represent the ‘seed banks’ of stress‐tolerant cells of different taxa, along with the cohort of photosynthetically active and actively growing but vulnerable‐to‐stress cells. However, this proposition requires further examination.

## 4 Conclusions

The photosynthetic activities of desert cyanobacterial and algal cells are highly variable rather than being constant. Only about a quarter of cyanobacteria and algae have relatively high photosynthetic activity, and these cells are, apparently, responsible for the bulk of CO_2_ fixation. Therefore, minor fluctuations in their abundance could significantly impact the total CO_2_ fixation in the desert. At least a third of all cyanobacteria and algae have low (< 20%) levels of CO_2_ fixation, and their proportion increases with aridity. Because the taxonomic compositions of the cyanobacterial and algal soil assemblages of the Negev Desert are typical for desert biocrusts, our findings could be justifiably extrapolated to deserts in other parts of the globe.

## Author Contributions

**Khin Maw Kyi:** methodology, investigation, visualization and formal analysis, writing the original draft, review and editing. **Mikhail V. Zubkov:** methodology, statistical analysis, writing the original draft, review and editing. **Nina A. Kamennaya:** conceptualization, funding acquisition, supervision, methodology, visualization, formal analysis and data curation, writing the original draft, review and editing.

## Supporting information


**Data S1:** ppl70634‐sup‐0001‐supinfo.pdf.

## Data Availability

The data that support the findings of this study is openly available in the OFS depository: https://doi.org/10.17605/OSF.IO/CH4SF.
