# Supplementary material for: A Minority of Desert Cyanobacteria and Algae Is Responsible for the Bulk of CO2 Fixation
Source: Physiol Plant. 2025 Nov 12;177(6):e70634. doi: 10.1111/ppl.70634 (PMC12611638; doi:10.1111/ppl.70634)
Supplement: Supplementary file 1 — Data S1: ppl70634‐sup‐0001‐supinfo.pdf. [file PPL-177-e70634-s001.pdf]

A minority of desert cyanobacteria and algae are responsible for the bulk of CO_2 fixation

Khin Maw Kyi, Mikhail V. Zubkov, Nina A. Kamennaya

TABLES AND FIGURES

SUPPLEMENTARY TABLE 1

Comparison of the geomean biovolume-specific CO_2 fixation rates (DPM µm^{-3}) of soil cyanobacteria and algae from the stream beds and slopes areas in the arid region in winter and spring and in the hyper-arid region in winter using the unbalanced two way ANOVA test of the logarithm-transformed rates. The unbalanced test was used because different number (*n*) of replicates comprised the data sets. For the arid region the three sampled areas, i.e., the stream bed, the south and north slopes, were chosen as the first factor and the two seasons, i.e., winter and spring, were chosen as the second factor. For the hyper-arid region the second factor was cyanobacteria versus algae, because measurements were made only in winter. The only statistically significant difference was between the geomean fixation rates of cyanobacteria and algae in the hyper-arid region in winter. The rest comparisons including interactions between the two factors were statistically insignificant. No difference was found between the geomean in the different areas sampled. Also no difference was found between the geomean in winter and spring for both cyanobacteria and algae.

Region			Geomean, DPM µm^{-3}				Unbalanced Two Way ANOVA	
Arid	Microorganism	Area	Winter	*n*	Spring	*n*	Factors	*P*-value
	Cyanobacteria	StreamBed	0.005915	10	0.004247	23	Streambed-South-North Slope	0.712
		SouthSlope	0.007278	9	0.003816	7	Winter vs Spring	0.1086
		NorthSlope	0.005989	9	0.006623	6	Interaction	0.3715
	Algae	StreamBed	0.002445	9	0.002009	21	Streambed-South-North Slope	0.09943
		SouthSlope	0.002641	7	0.000828	8	Winter vs Spring	0.2811
		NorthSlope	0.00249	9	0.003703	8	Interaction	0.07382
Hyperarid		Area	Cyanobacteria	n	Algae	n	Factors	P-value
		Streambed	0.002389	2	0.001768	10	Streambed vs Slope	0.8802
		Slope	0.004887	20	0.001425	8	Cyanobacteria vs Algae	**0.003816**
							Interaction	0.2463

The difference was considered statistically significant when $P < 0.05$, marked in bold.

SUPPLEMENTARY TABLE 2

Comparison of the mean diameter (µm) of algal cells and mean width (µm) of cyanobacterial filaments between the different batches of samples from the arid region in winter and spring and from the hyper-arid region in winter using One-Way ANOVA test followed by Tukey HSD / Tukey Kramer test. There is no significant difference between the means of any pair.

**Algae**	Batch 1	Batch 2	Batch 3	Batch 4	Batch 5	Batch 6
n	109	108	73	98	39	108
***P*-value**						
Batch 2	0.0765					
Batch 3	0.2564	0.9998				
Batch 4	0.9868	0.3564	0.6455			
Batch 5	0.1856	0.9992	0.9946	0.4589		
Batch 6	0.6605	0.8401	0.9655	0.9649	0.8229	

**Cyanobacteria**	Batch 1	Batch 2	Batch 3	Batch 4	Batch 5
n	81	83	97	35	87
***P*-value**					
Batch 2	0.9823				
Batch 3	0.9503	0.9999			
Batch 4	0.5639	0.3042	0.2255		
Batch 5	0.9516	0.7026	0.5668	0.8734	

The difference was considered statistically significant when $P < 0.05$.

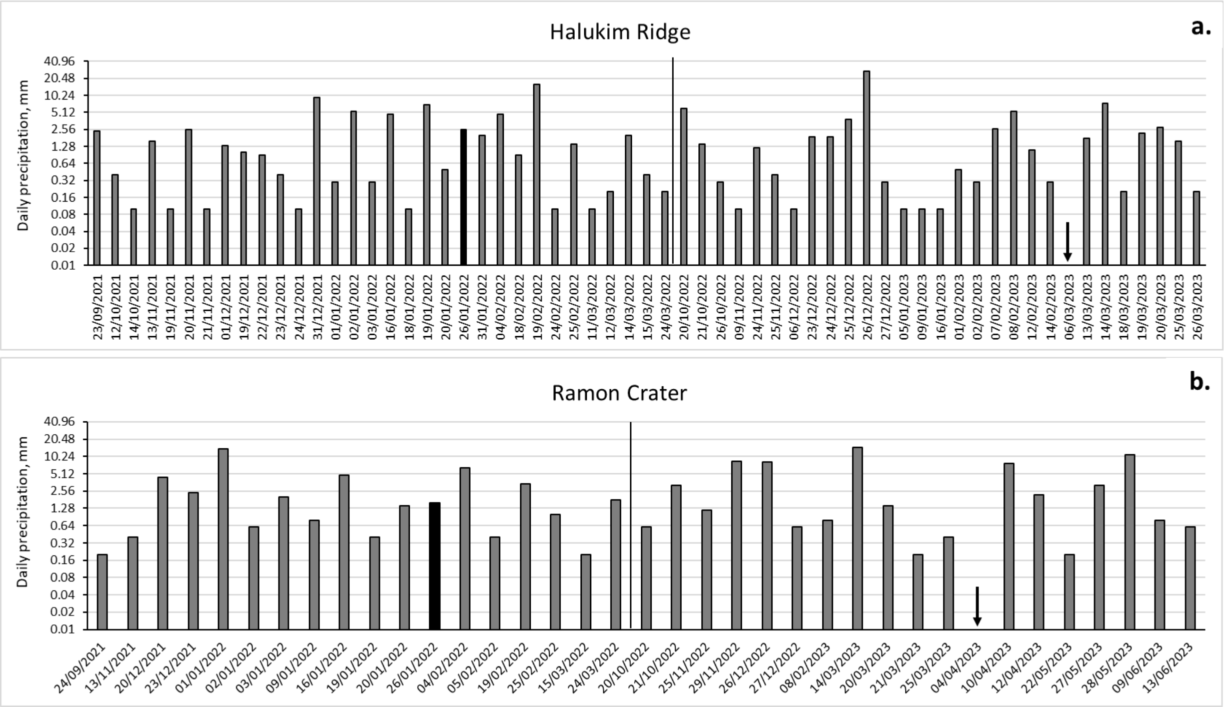

SUPPLEMENTARY FIGURE 1

Daily precipitation (mm) at the **a**) hyper-arid Ramon Crater and **b**) arid Halukim Ridge in the Central Negev Desert. Only rainy days are shown. Black bars indicate the date of sample collections after rain in winter 2022. Arrows indicate the date of sample collection after dewfall in spring 2023.

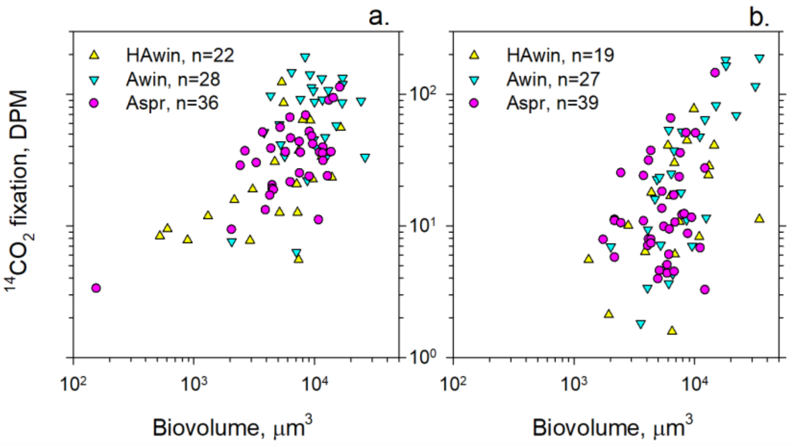

SUPPLEMENTARY FIGURE 2

The amount of ^{14}C-labeled CO_2 assimilated per cellular biovolume of **a**) cyanobacteria or **b**) algae from soil collected from a hyper-arid region in winter and from an arid region in winter and spring.

Because the profiles had different number of points (Supplementary Figure 2a, b), we used the ranks of the profile with less points as a template to match the closest ranks of the profile with more points (Supplementary Figure 3). After that we calculated the difference between the two matched profiles by subtracting the percentages of the lower profile from the percentages of the higher profile. The results are presented as box plots (Supplementary Figure 3 inserts). The Null Hypothesis that the differences do not differ from zero was tested using the single-sample *t*-test (Supplementary Figure 3).

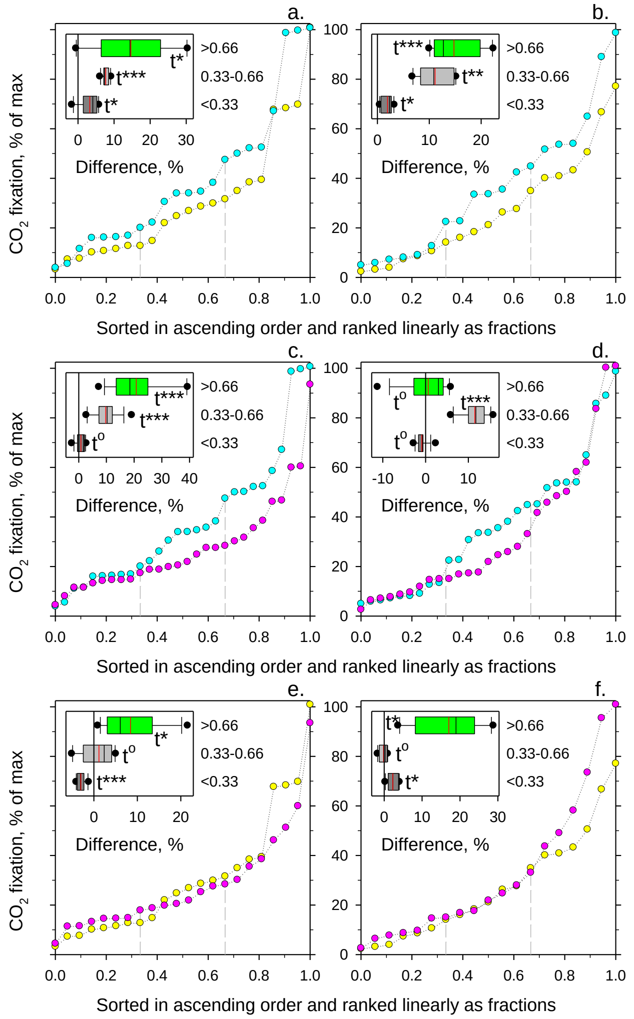

SUPPLEMENTARY FIGURE 3

Paired comparison of relative biovolume-specific CO_2 fixation of soil **a**) cyanobacteria and b) algae collected in winter from the hyper-arid (yellow) and arid (cyan) regions; of soil **c**) cyanobacteria and **d**) alga collected from the arid region in winter (cyan) and spring (pink); of soil **e**) cyanobacterial and **d**) algal collected from the hyper-arid region in winter (yellow) and arid region in spring (pink). The inserted box plots show the differences between the paired profiles for the three activity parts, i.e., <0.33 rank (dark grey), 0.33-0.66 ranks (light grey) and >0.66 rank (green) boxes. The results of the corresponding single-sample *t*-test are t^0 – $P > 0.05$; t* - $0.0005 < P < 0.005$; t** - $0.00005 < P < 0.0005$; t*** - $P <$ 0.00005.

Comparison of the cyanobacterial and algal winter profiles from the hyper-arid and arid regions (Supplementary Figure 3a, b) showed that both types of microorganisms were photosynthetically significantly more active in the arid region, although the differences among cyanobacteria and algae in the low-activity part were minor, within few percent.

Comparison of the profiles from the arid region sampled in winter and spring (Supplementary Figure 3c, d) revealed no statistically significant differences among cyanobacteria and algae in the low-activity part. This suggests that a third of cells maintain low photosynthetic activity along the entire rainy season when most cellular growth of desert cyanobacteria and algae is expected. In the middle-activity part both cyanobacteria and algae were photosynthetically significantly more active in winter than in spring, whilst in the high-activity part the activity in winter was significantly higher for cyanobacteria only.

Comparison of the profiles from the hyper-arid region in winter and arid region in spring (Supplementary Figure 3e, f) revealed no statistically significant differences among cyanobacteria and algae in the medium-activity part only. In the low-activity part of the profile both cyanobacteria and algae were more active in the arid region, whilst in the high-activity part cyanobacteria were more active in the hyper-arid region whereas algae were more active in the arid region.

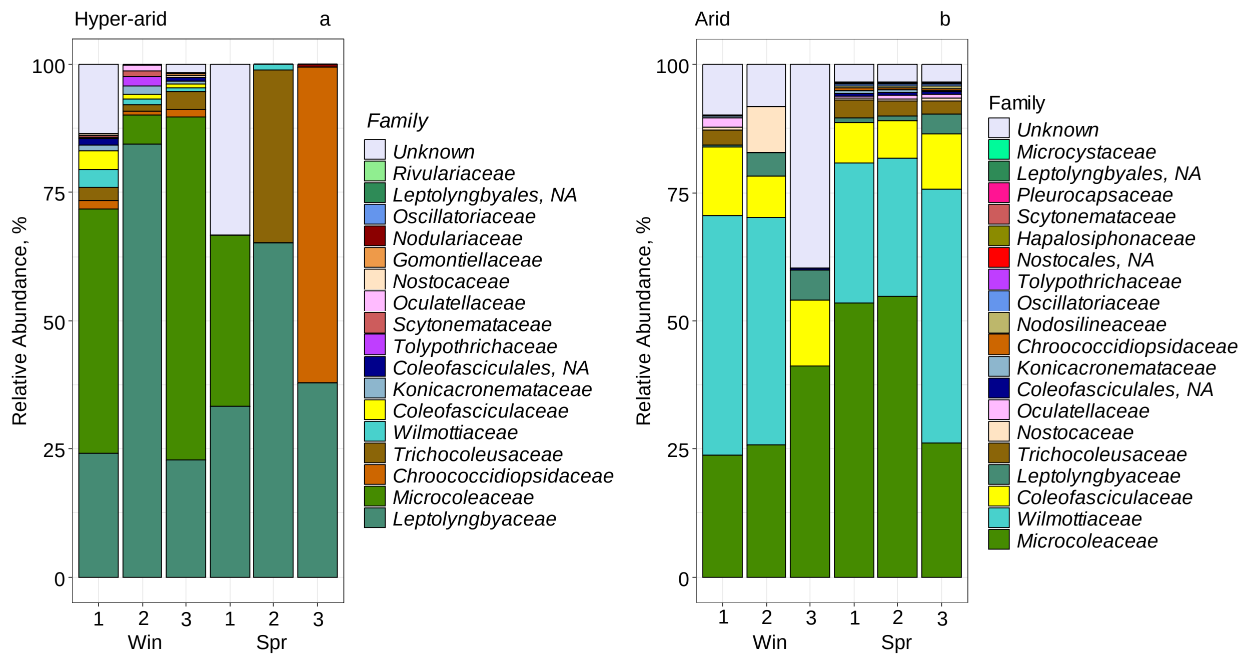

SUPPLEMENTARY FIGURE 5

Relative abundance of cyanobacterial families in soil collected from **a**) hyper-arid and **b**) arid regions in winter (Win) and spring (Spr).
